# Structure-Based Design of Porcine Circovirus Type 2 Chimeric VLPs (cVLPs) Displays Foreign Peptides on the Capsid Surface

**DOI:** 10.3389/fcimb.2018.00232

**Published:** 2018-07-09

**Authors:** Dongliang Wang, Sujiao Zhang, Yawen Zou, Wanting Yu, Yifan Jiang, Yang Zhan, Naidong Wang, Yanpeng Dong, Yi Yang

**Affiliations:** ^1^Hunan Provincial Key Laboratory of Protein Engineering in Animal Vaccines, Laboratory of Functional Proteomics, Research Center of Reverse Vaccinology, College of Veterinary Medicine, Hunan Agricultural University, Changsha, China; ^2^Jiangsu Nannong Hi-Tech Co., Ltd, Jiangyin, China

**Keywords:** porcine circovirus type 2 (PCV2), chimeric virus-like particles (cVLPs), bivalent or multivalent vaccines, capsid protein, 3D structure prediction

## Abstract

Although porcine circovirus-like particles can function as a vector to carry foreign peptides into host cells, displaying foreign peptides on the surface of virus-like particles (VLPs) remains challenging. In this study, a plateau, consisting of the middle portion of Loop CD (MP-Lcd) from two neighboring subunits of PCV2 capsid protein (Cap), was identified as an ideal site to insert various foreign peptides or epitopes and display them on the surface of PCV2 VLPs. One of the goals of this work is to determine if the surface pattern of this plateau can be altered without compromising the neutralizing activity against PCV2 infections. Therefore, biological roles of MP-Lcd regarding VLPs assembly, cell entry, and antigenicity were investigated to determine whether this was a universal site for insertion of foreign functional peptides. Three-dimensional (3D) structure simulations and mutation assays revealed MP-Lcd was dispensable for PCV2 Cap assembly into VLPs and their entry into host cells. Notably, substitution of MP-Lcd with a foreign peptide, caused surface pattern changes around two-fold axes of PCV2 VLPs based on 3D structure simulation, but was not detrimental to VLPs assembly and cell entry. Moreover, this substitution had no adverse effect on eliciting neutralizing antibodies (NAbs) against PCV2 infection in pigs. In conclusion, MP-Lcd of the PCV2 Cap was a promising site to accommodate and display foreign epitopes or functional peptides on the surface of PCV2 VLPs. Furthermore, chimeric VLPs (cVLPs) would have potential as bivalent or multivalent vaccines and carriers to deliver functional peptides to target cells.

## Introduction

Porcine circovirus (PCV), a member of the *Circovirus* genus in the *Circoviridae* family, has a small, single-stranded and circular DNA genome. There are two main genotypes, namely porcine circovirus type 1 (PCV1) and PCV2, both of which share high levels of nucleotide homology and a common genomic organization (Segales et al., 2013). PCV2, the key causative agent of porcine circovirus-associated diseases (PCVADs), causes severe economic losses worldwide. Based on genome or *cap* gene (it encodes the sole structural protein of PCV2 capsid) analysis of PCV2 isolates, PCV2 is divided into four main subtypes (PCV2a, PCV2b, PCV2c, and PCV2d) (Franzo et al., 2015). Furthermore, PCV2a is subdivided into five clusters (2A, 2B, 2C, 2D, and 2E), PCV2b into three clusters (1A, 1 B, and 1C), whereas PCV2c has only been reported in Denmark, with only three isolates in GenBank (An et al., 2007; Olvera et al., 2007). Recently, a novel PCV genotype was identified (Phan et al., 2016; Palinski et al., 2017; Zhang et al., 2017) and temporarily designated PCV3, as its genomic DNA only shared ~30% nucleotide identities with PCV1 and PCV2 (Palinski et al., 2017). However, pathogenicity and potential lesions caused by PCV3 remain to be determined.

The circular genome of PCV2 contains 1766–1768 nucleotides, encoding two main viral proteins: the capsid protein (Cap) and virus replication associated proteins (Rep and its isoform of Rep', formed via alternative splicing) (Cheung, 2003). The PCV2 Cap, the sole structural protein of this virus, is composed of ~233 residues, with 60 of the Caps capable of self-assembly into a virus-like particle (VLP) *in vitro* (Khayat et al., 2011). Furthermore, the VLP has been successfully used as the main antigen in commercial vaccines against PCV2 infection (Fachinger et al., 2008). In addition, PCV2 VLPs instead of virus, were exploited to study interaction of virus-host cells (Misinzo et al., 2006) and mechanism of virus entry into various swine host cell lines (i.e., PK15 and 3D/4 cell lines) (Misinzo et al., 2005, 2009).

The three-dimensional (3D) structure of the PCV2 Cap was elucidated by two research groups (Khayat et al., 2011; Liu et al., 2016). The Cap contains a typical jelly-roll fold composed of eight β-strands, connected via seven loops (Khayat et al., 2011). There are indications that these loops are responsible for assembly of VLPs and that they determine surface patterns of the PCV2 capsid (Khayat et al., 2011; Wang et al., 2016). Further, based on 3D structure analyses, loops exposed on the outer surface of the capsid have substantially diverged during PCV2 evolution (Wang et al., 2016). In addition, both the NH_2_- and carboxyl-terminus (NT & CT) of the Cap contain a non-structured stretch. The NT, rich in basic amino acids, has a typical nuclear localization signal (NLS), responsible for distribution of the PCV2 Cap in nucleus (Liu et al., 2001) and viral genome packaging (Khayat et al., 2011). Therefore, in a stable PCV2 capsid, the NT is located inside the capsid and interacts with viral genomic DNA, whereas the CT is exposed on the surface of the capsid, as illustrated in 3D structures (Khayat et al., 2011; Liu et al., 2016; Wang et al., 2016). Although both termini of the Cap have been engineered as targeted sites for insertions of or substitutions with foreign epitopes or functional peptides to generate chimeric VLPs (cVLPs) or chimeric PCV, foreign peptides located at the NT of the Cap may hide within PCV2 VLPs and consequently may not be displayed on the exterior surface of the capsid if the NT is not externalized. Chimeric PCV were constructed by insertion of various epitopes or tags into the 3'-terminal end of the *cap* gene (the CT of the Cap), and specific antibodies against these tags or foreign peptides were produced in response to these chimeric PCVs (Beach et al., 2011; Piñeyro et al., 2015a,b). Furthermore, maximal insertion size of foreign amino acid residues at the 3'-terminal end of the *cap* gene has also been studied. Chimeric porcine circovirus (PCV) containing amino acid epitope tags in the C terminus of the capsid gene are infectious and elicit both anti-epitope tag antibodies and anti-PCV type 2 neutralizing antibodies in pigs (Beach et al., 2011).

Our laboratory previously designed and prepared PCV2 cVLPs to display a foreign epitope on the exterior surface of the cVLPs, based on 3D structural analyses of the PCV2 Cap and its assembly of the capsid (Hu et al., 2016). Several loops were considered for insertions of the GP5 epitope B of porcine reproductive and respiratory syndrome virus (PRRSV), although only Loop CD was an ideal site for insertion of the GP5 epitope B, since this insertion had no negative effects on either assembly or cell entry of cVLPs *in vitro*.

In this study, a plateau consisting of MP-Lcd around two-fold axes of PCV2 VLPs was used to determine, based on 3D structure analyses and simulations, its potential as a universal platform for insertions of various foreign peptides without compromising assembly and cell entry of PCV2 cVLPs. Notably, roles of MP-Lcd in PCV2 VLPs assembly, cell entry and antigenicity were evaluated.

## Materials and methods

### Reagents, genes, plasmids, and cell lines

Unless otherwise stated, all chemicals were purchased from Sigma Aldrich (St. Louis, MO, USA). PCV2 *cap* gene (GenBank accession number: JF504708) was optimized and synthesized, as we have described (Hu et al., 2016). Mutated PCV2 cap genes were constructed by overlapping polymerase chain reaction (PCR), as described (Hu et al., 2016). Resultant DNA fragments were subcloned into a pET100 vector (Invitrogen, Carlsbad, CA, USA; **Figure 2A**). After confirmation by DNA sequencing, recombinant plasmids were transformed into BL21 (DE3) competent cells (TransGen, Beijing, China) for protein expression.

### PCV2 cap expression, purification, and self-assembly of VLPs *in vitro*

Protein expression, purification, and VLPs assembly *in vitro* of PCV2 Cap wild type and its mutants were done as described (Zhang et al., 2016). For western blots, proteins from cell lysates were first separated by 10% sodium dodecyl sulfate polyacrylamide gel electrophoresis (SDS-PAGE) (Bio-Rad, Hercules, CA, USA). Thereafter, proteins were transferred to a polyvinylidene difluoride membrane (PVDF; Life Technologies, Carlsbad, CA, USA) in a protein transfer device (Bio-Rad) at 80 V for 1 h. The membrane was then blocked with 3% bovine serum albumin (BSA; Roche, Basel, Switzerland) in phosphate-buffered saline (1 × PBS, pH 7.4) for 1 h, and then primary antibody (rabbit anti-PCV2 Cap, 1:500) was incubated with the membrane overnight. A horseradish peroxidase (HRP)-conjugated goat anti-rabbit IgG (1:5,000, Promega, Madison, WI, USA) was the secondary antibody and signals were visualized with High-sig ECL western blotting substrate (Tanon, Shanghai, China). Formation of VLPs was confirmed by transmission electron microscopy (TEM). The PCV2 VLPs were adsorbed on to carbon-coated copper grids for 10 min and stained with 1% phosphotungstic acid for 10 min. Subsequently, VLPs were examined with TEM (CM100, Philips Electron Optics, Zurich, Switzerland).

### Cap sequences alignment and 3D structure simulations of PCV2 Cap and capsid

Typical Cap sequences from PCV1, 2, and 3 were downloaded from GenBank (http://www.ncbi.nlm.nih.gov/). Subtype and cluster of each PCV2 isolate were confirmed via previous phylogenetic analyses (Zhan et al., 2016). Amino acid sequences were aligned using a web server (http://multalin.toulouse.inra.fr/multalin/). For each Cap mutant, a 3D structural model was generated via homology modeling using a crystal structure of PCV2 Cap (PDB accession number: 3R0R) as a template, with protein modelings done using Modeller (http://salilab.org/modeller/). Then, icosahedral structures (3D) of all capsid mutants were generated, based on the monomeric structure of the PCV2 Cap mutant, by applying a VMD 1.9 matrix transformation using Tcl script (Humphrey et al., 1996) and displayed with PyMol (Version 1.8.4.0, http://www.pymol.org/).

### Preparation of anti-PCV2 Cap sera

Female New Zealand rabbits (*n* = 2), 2 mo old, were used to prepare anti-PCV2 Cap serum. Rabbits were initially immunized with 2 ml of PCV2 VLPs (wild type; 0.2 mg/ml) mixed with Alhydrogel adjuvant (InvivoGen, San Diego, CA, USA), and then similarly boosted twice more at 2-week intervals (Weeks 2 and 4). Blood samples were collected 6 wk after final immunization and serum was isolated and stored at −20°C.

### Indirect immunofluorescence assays (IFAs)

Cell entry of PCV2 VLPs were detected with IFAs, as described (Misinzo et al., 2005). PK15 cells cultured in 24-well plates were incubated with 2 μg of PCV2 VLPs in each well for 1 h. After incubation, PK15 cells were washed three times with PBS to remove unbound VLPs. As a control, PBS without VLPs was added to PK15 cells. For immunofluorescent staining, PK15 cells were fixed in 4% (wt/vol) paraformaldehyde in PBS at12 h post-inoculation of PCV2 VLPs. Cells were subsequently washed and permeabilized with 0.1 % Triton X-100 for 5 min at room temperature. Cells were washed three times with PBS, incubated with rabbit anti-PCV2 serum (1:1,000) for 1 h, and washed three times with PBS. Then, cells were incubated with fluorescein isothiocyanate (FITC) conjugated donkey anti-rabbit IgG (1:2,000, Life Technologies) for 1 h in the dark, and subsequently washed three times. Finally, cells were mounted with Prolong® Gold Anti-fade Reagent with 4′,6-diamidino-2- phenylindole (DAPI; Invitrogen) for nucleic acid staining and imaged with confocal microscopy (LSM 710 NLO & DuoScan System, Carl Zeiss, Germany).

### Immunization and serum collection

Fifteen commercial 3-week-old pigs, serologically negative for PCV2 and porcine reproductive and respiratory syndrome virus (PRRSV), were used. These pigs were randomly allocated into three groups (five per group). Groups 1 and 2 were immunized with intramuscular injections of 200 μg of PCV2 wild type VLPs or cVLPs-M8 (**Figure 2A**) mixed with MontanideTM Gel 01 ST adjuvant (SEPPIC), respectively, whereas Group 3 was immunized with PBS only (negative control). Each pig was injected only once. Sera were collected 21 and 28 d post-primary immunization (dpi) and tested by indirect enzyme-linked immunosorbent assay (ELISA) and PCV2 neutralization assays. All animal care and use protocols in this study were conducted in accordance with the guidelines of the Animal Care and Use Committee of Hunan Agricultural University and were approved by the Institutional Ethics Committee (IEC) of Hunan Agricultural University.

### Indirect ELISA and PCV2 neutralization assay

PCV2 VLPs assembled from wild type PCV2 Cap (Cap WT) were coated as an antigen to detect specific anti-PCV2 antibodies by ELISA, as described (Zhang et al., 2016). The PCV2 neutralization assay was also performed as described (Fort et al., 2007). All serum samples from each group were heat-inactivated (56°C for 30 min) and 50 μl of inactivated sera was serially two-fold diluted with PBS, and then mixed with 50 μl of PCV2 virus (200 TCID50; GenBank accession number: KP112484) for 1 h at 37°C. Then, serum-virus mixtures were inoculated to confluent PK-15 cells cultured in 96-well plates and incubated at 37°C for 72 h. Cells were fixed with absolute ethyl alcohol for 30 min, 0.1% triton for 10 min and then blocked with 3% BSA for 1 h at room temperature. Thereafter, cells were incubated with rabbit anti-PCV2 serum (1:1,000) as the primary antibody and FITC-conjugated donkey anti-rabbit IgG (1:2,000 dilution, Life Technologies) as second antibody, each for 1 h. Cells were observed using a fluorescent microscope (Olympus IX73, Tokyo, Japan). The reciprocal of the highest dilution at >50% fluorescent focus reduction was the neutralizing antibody titer.

## Results

### MP-Lcd from two neighboring Cap subunits formed a plateau on the two-fold axis of PCV2 capsid

**Figure 1:**
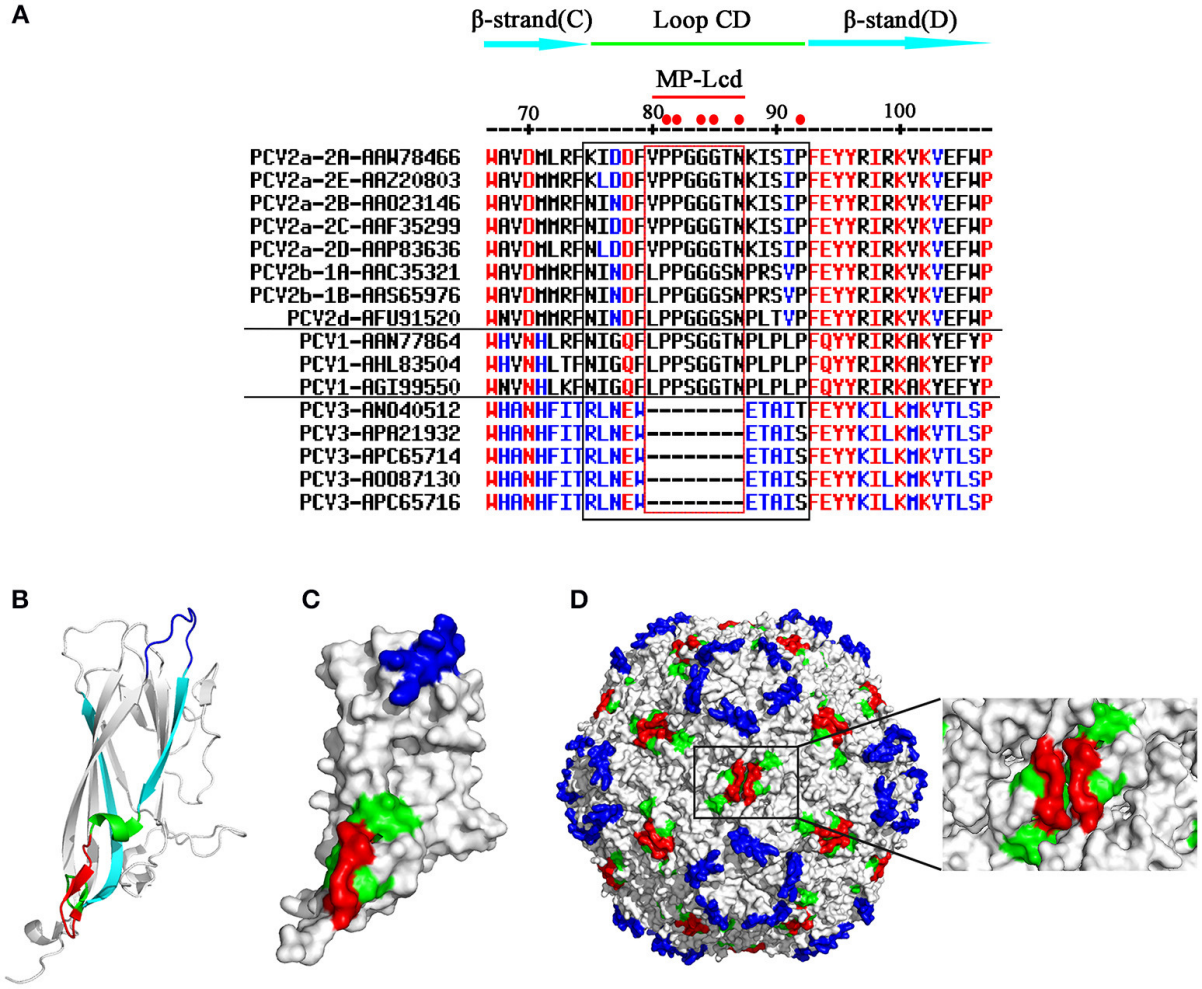
Primary and 3D structures of Loops CD among PCV Caps. **(A)** Comparative sequence alignments of β-stand C, Loop CD, and β-stand D among various PCV Caps (top). Numbers on the top indicate positions of residues in PCV2 Caps. Genotype, subtype, and GenBank accession number of each sequence are shown in the left column. A *cap* gene derived from subtype of PCV2b-1B was used for this study. Residues within the black rectangle represent Loops CD, whereas residues within the red rectangle indicate MP-Lcd. Red dots on the top indicate residues conserved between PCV1 and PCV2 Caps. Hyphen (–) means residue is missing at the indicated position. **(B,D)** 3D localizations of MP-Lcd on PCV2 Cap backbone **(B)**, PCV2 Cap surface **(C)**, and PCV2 capsid **(D)**. Data of 3D structure of PCV2 Cap and capsid were retrieved from Protein Data Bank (http://www.rcsb.org/; PDB accession number: 3R0R). MP-Lcd was labeled in red; β-strands C and D were labeled in cyan and Loop CD (except MP-Lcd) was labeled in green. Loop BC colored in blue demonstrates surfaces of icosahedral five-fold axes of PCV2 capsid.

**Figure 2:**
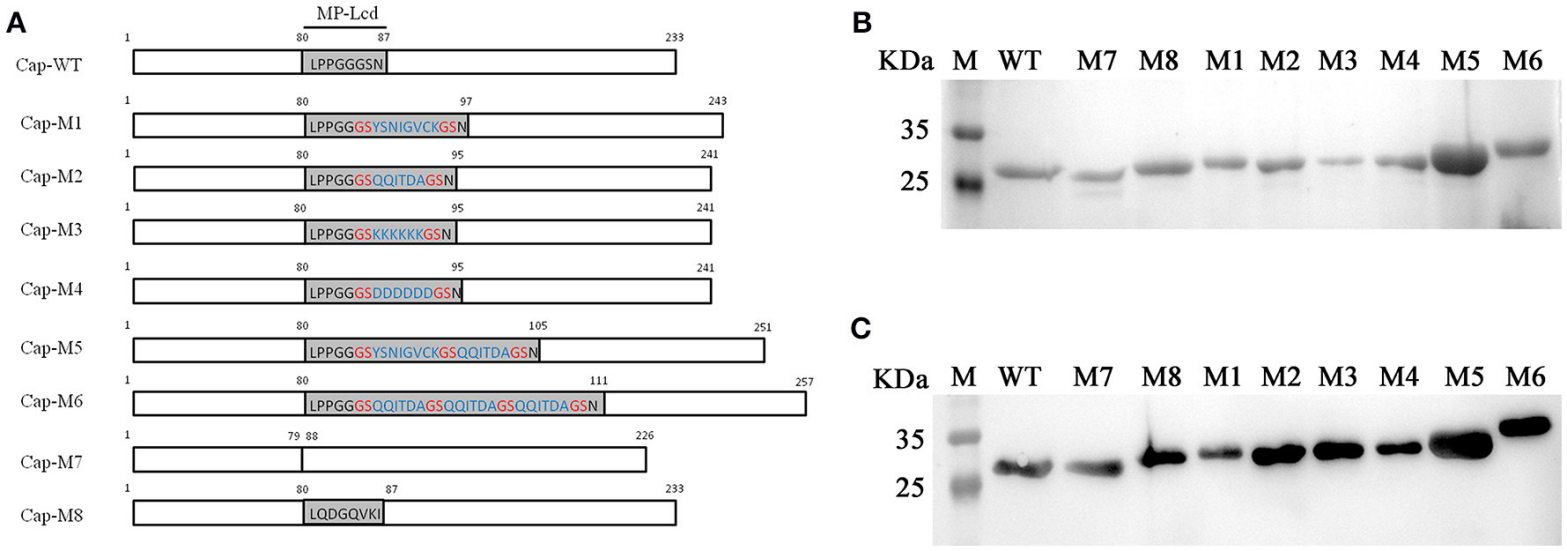
PCV2 Cap WT and mutants. **(A)** Residues within MP-Lcd of PCV2 Cap WT and mutants. Numbers indicate residue positions in PCV2 Cap and mutants. Sequence of MP-Lcd is shown in Cap-WT. Residues in blue represent foreign peptides inserted into MP-Lcd of PCV2 Cap, whereas residues in red indicate “GS” linkers between foreign peptides or foreign peptides and PCV2 Cap. Note: MP-Lcd is substituted by an epitope derived from PEDV spike protein in Cap-M8. **(B)** SDS-PAGE of purified Cap WT and Cap mutants via Ni-NTA affinity chromatography. Lane M, protein marker; expected recombinant Cap protein is indicated in each lane. **(C)** Western blots of supernatants from bacterial lysates containing expressed Cap-WT and various mutants. Lane M, protein marker; the expected specific band is present in each lane.

Loop CD, connecting β-strands C and D in PCV Cap, was a highly divergent loop among distinct genotypes of porcine circoviruses (PCV1, PCV2, and PCV3), based on amino acid sequence alignment (Figure 1A). There was no strictly conserved residue present in this loop among the three genotypes (Figure 1A). In contrast, 6 of 18 residues (81PP82, 84GG85, 87N, and 92P) between Caps of PCV1 and 2 were invariant (solid red dots at top of Figure 1A). In addition, 5 of the 6 conserved residues were located within the middle portion (eight residues from positions 80–87; red rectangle in Figure 1A) of Loop CD. Therefore, the portion composed of these eight residues was designated MP-Lcd (Middle Portion of Loop CD). In the 3D structure, Loop CD of the PCV2 Cap formed the second elevation on the exterior surface of the PCV2 capsid, with most residues in Loop CD located on surfaces of both the PCV2 Cap and the capsid (Figures 1B–D) (Khayat et al., 2011; Wang et al., 2016), whereas MP-Lcd formed a plateau on the surface of the two-fold axes of the PCV2 capsid (red portions in Figure 1D). Of note, MP-Lcd was absent among PCV3 Caps (Figure 1A). Based on structural features and absence of MP-Lcd in PCV3 Cap, together with previous study that an epitope derived from PPRSV GP5 was successfully inserted Loop CD (between 85G and 86S of PCV2 Cap) (Hu et al., 2016). we inferred that MP-Lcd was an optimal target site for inserting foreign peptides and displaying them on the surface of the PCV2 capsid. To confirm this hypothesis, a series of PCV2 Cap mutants (Cap-M1-6 in Figure 2A) were designed to test tolerances of MP-Lcd to foreign peptides.

### MP-Lcd had high tolerances to insertions of various foreign peptides

**Figure 3:**
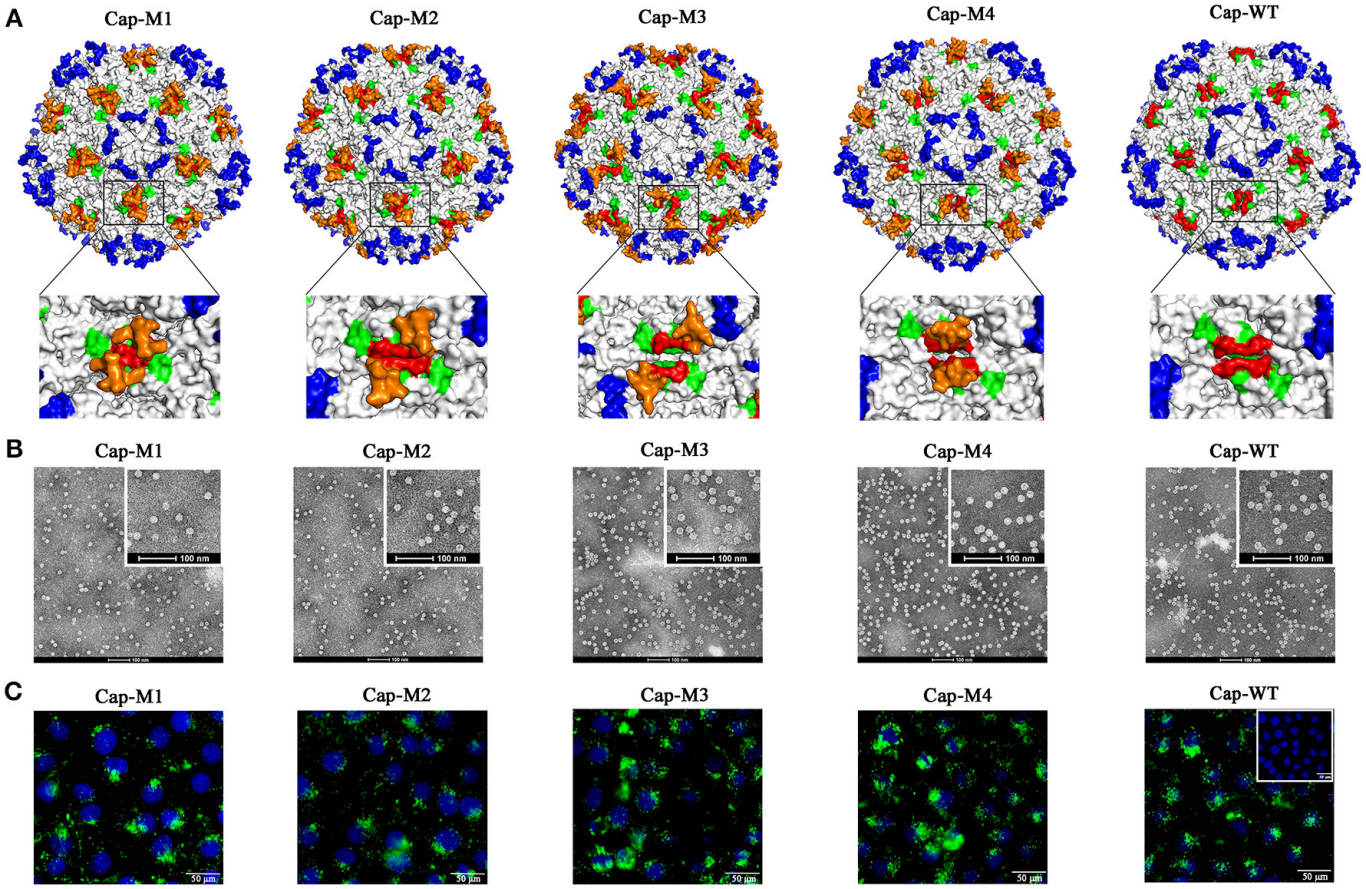
PCV2 VLPs assembly and cell entry into PK15 cells. **(A)** Localizations of foreign peptides or epitopes on the surface of PCV2 capsid in simulated 3D models after assembly of PCV2 Cap-WT and mutants into VLPs. Images on bottom of **(A)** demonstrate (high magnification) 3D structures or orientations of foreign epitopes and artificial peptides on a plateau formed by two neighboring MP-Lcd. Foreign epitopes or artificial peptides were labeled in orange; MP-Lcd was labeled in red and Loop CD, except MP-Lcd was labeled in green and Loop BC-decorated five-fold axes of the icosahedra were labeled in blue on PCV2 capsid. **(B)** PCV2 VLPs formations observed by TEM. Negative staining of VLPs assembled from PCV2 Cap WT or mutants (bars = 100 nm). **(C)** Entry of PCV2 VLPs into PK-15 cells. Internalizations of PCV2 VLPs assembled from PCV2 Cap WT or Cap mutants (M1-M4) were confirmed by confocal microscopy. Inset in PCV2 Cap-WT represents a negative control in which PBS instead of VLPs was added into PK15 cell culture. Green fluorescence represents PCV2 Cap in PK15 cells. Nuclei (blue) of PK-15 cells were stained by DAPI.

Four peptides, including two epitopes derived from structural proteins of porcine epidemic diarrhea virus (PEDV) (Sun et al., 2008) and porcine parvovirus (PPV) (Sun et al., 2015), respectively (Figure 2A, Cap-M1 and -M2), and two artificial peptides (Figure 2A, Cap-M3 and -M4), were designed. Based on simulated 3D structures, these four peptides projected from a plateau formed by the MP-Lcd formed (orange in Figure 3A) after they were inserted into the PCV2 Cap. To test effects of insertions of various peptides in MP-Lcd on VLPs assembly and cell entry, Cap mutants (Cap-M1 to -M4, Figure 2A) were successfully expressed and purified (Figures 2B,C). Of note, these Cap mutants were capable of self-assembling into VLPs after *in vitro* dialysis (Figure 3B). Moreover, insertion of various peptides into MP-Lcd had no adverse effect on entry of these VLPs into PK15 cells (Figure 3C). Therefore, MP-Lcd tolerated insertions with various foreign peptides (Cap-M1 to 4), including those with highly charged residues (Cap-M3 and 4).

### Capacity for MP-Lcd to accommodate foreign peptides

**Figure 4:**
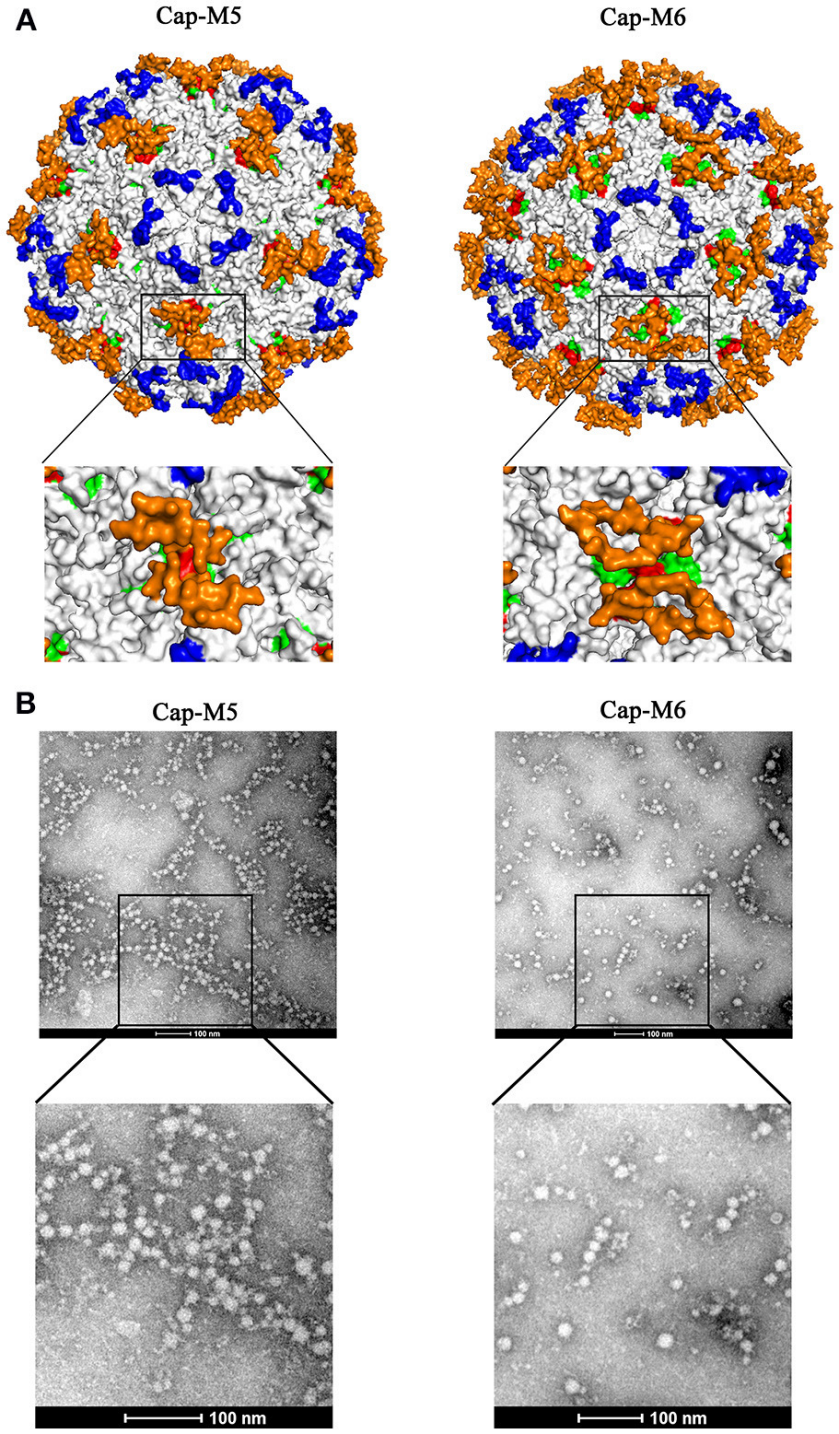
Insertions of large fragments had an adverse effect on PCV2 VLPs assembly. **(A)** Simulated 3D structures of foreign epitopes (Cap-M5 and M6) on the surface of the PCV2 capsid. Images on bottom of **(A)** show top views (high magnification) of foreign epitopes on a plateau formed by two neighboring MP-Lcd. Foreign epitopes were labeled in orange; MP-Lcd was labeled in red and Loop CD, except MP-Lcd was labeled in green and Loop BC-decorated five-fold axes of the icosahedra were labeled in blue on PCV2 capsid. **(B)** Morphologies of PCV2 Cap-M5 and -M6 after dialysis against assembly buffer. Morphologies were observed by TEM after samples were negatively stained.

Having established that MP-Lcd allowed insertions of various foreign peptides without compromising *in vitro* assembly of VLPs and entry into PK15 cells, maximal insertion size was studied. Two new Cap mutants were designed. One contained an insertion of two distinct epitopes (derived from structural proteins of PEDV and PPV) spaced by a “GS” linker (Cap-M5, Figure 2A), whereas another had an insertion of three repeats of the epitope derived from PPV, also spaced by two “GS” linkers (Cap-M6, Figure 2A). The simulated 3D structures demonstrated that both foreign peptides might be displayed on the outer surface of PCV2 VLPs (Figure 4A). In addition, both Cap mutants were also successfully purified after being expressed in bacteria (Figures 2B,C). After the two mutants were dialyzed against assembly buffer *in vitro*, morphologies were distinct from those of VLPs assembled from either the Cap WT or other Cap mutants (Figure 4B). Based on TEM results, we inferred that PCV2 VLPs assembly may have been adversely affected by 18 residues, in the absence of further optimization of linker or assembly conditions.

### MP-Lcd was dispensable for PCV2 VLPs assembly and cell entry

**Figure 5:**
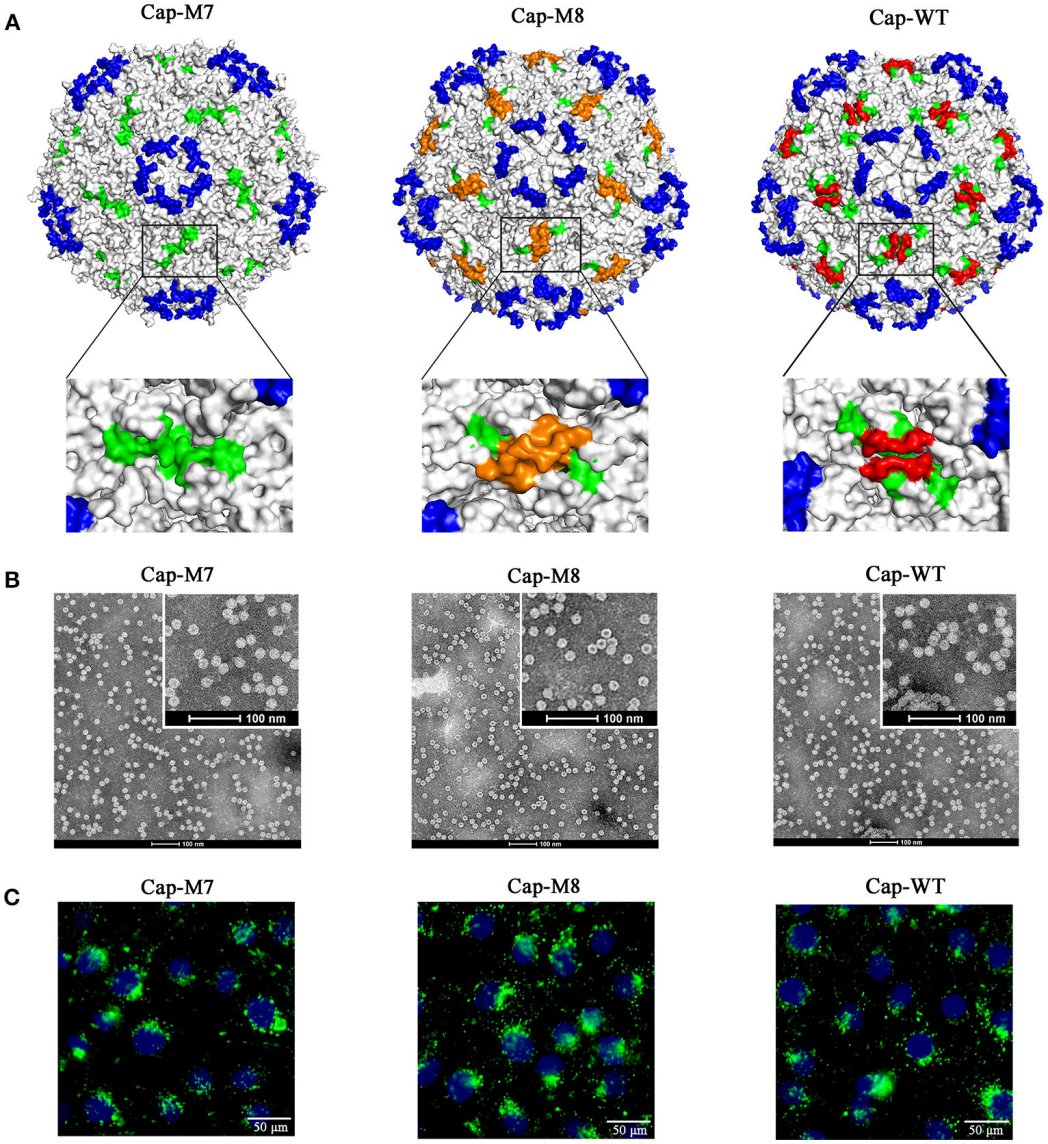
MP-Lcd was not required for PCV2 VLPs assembly and entry into PK15 cells. **(A)** Surface alterations around two-fold axes of PCV2 icosahedra due to ML-Lcd deletion (Cap-M7) or replacement by a foreign epitope (Cap-M8) in simulated 3D models. Magnified images (top view) around a two-fold axis of PCV2 icosahedra were shown on bottom of **(A)**. Loop CD, except MP-Lcd was colored in green; the foreign epitope was colored in orange; MP-Lcd was colored in red, whereas blue indicated Loop BC-decorated five-fold axes of the PCV2 icosahedra. **(B)** Removal of MP-Lcd or replacing it with a foreign epitope had minimal effect on assembly of VLPs. Morphologies of PCV2 VLPs assembled from the Cap-M7, -M8, or WT, respectively were confirmed by TEM (bars = 100 nm). **(C)** Entry of PCV2 VLPs into PK-15 cells. Internalizations of PCV2 VLPs assembled from PCV2 Cap-M7, -M8, or WT were confirmed by confocal microscopy. Green fluorescence represented PCV2 Cap in PK15 cells. Nuclei (blue) of PK-15 cells were stained by DAPI.

To test functions of MP-Lcd on PCV2 VLPs assembly *in vitro* and cellular uptake, we designed two PCV2 Cap mutants (Cap-M7 and -M8 in Figure 2A), of which MP-Lcd was either deleted (M7) or replaced (M8) by a foreign epitope derived from a structural protein of PEDV spike protein (Sun et al., 2008). The 3D structure simulations revealed both PCV2 Cap mutants had a similar jelly-roll fold as wild type **(Figure 5A**). However, when Cap-M7 was assembled into a capsid, the plateaus composed of MP-Lcd around the two-fold axes had disappeared (Figure 5A). In contrast, a new surface pattern around the two-fold axes was formed in a capsid assembled by Cap-M8 (Figure 5A), due to replacement of MP-Lcd with the foreign epitope. Further, both mutants were expressed and purified via NTA-Ni2+ chromatography (Figures 2B,C). After dialysis *in vitro*, both were capable of assembling into VLPs with a diameter of ~17 nm, as confirmed by TEM (Figure 5B), with very similar morphology as VLPs assembled from the WT of the Cap (Figure 5B). Based on IFA, VLPs assembled from both Cap mutants entered PK15 cells similar to wild type (Figure 5C). Therefore, MP-Lcd appeared dispensable for VLPs assembly *in vitro* and subsequent entry into PK15 cells.

### Replacement of MP-Lcd with a foreign epitope had minimal effect on PCV2 antigenicity and production of PCV2-specific NAbs in pigs

**Figure 6:**
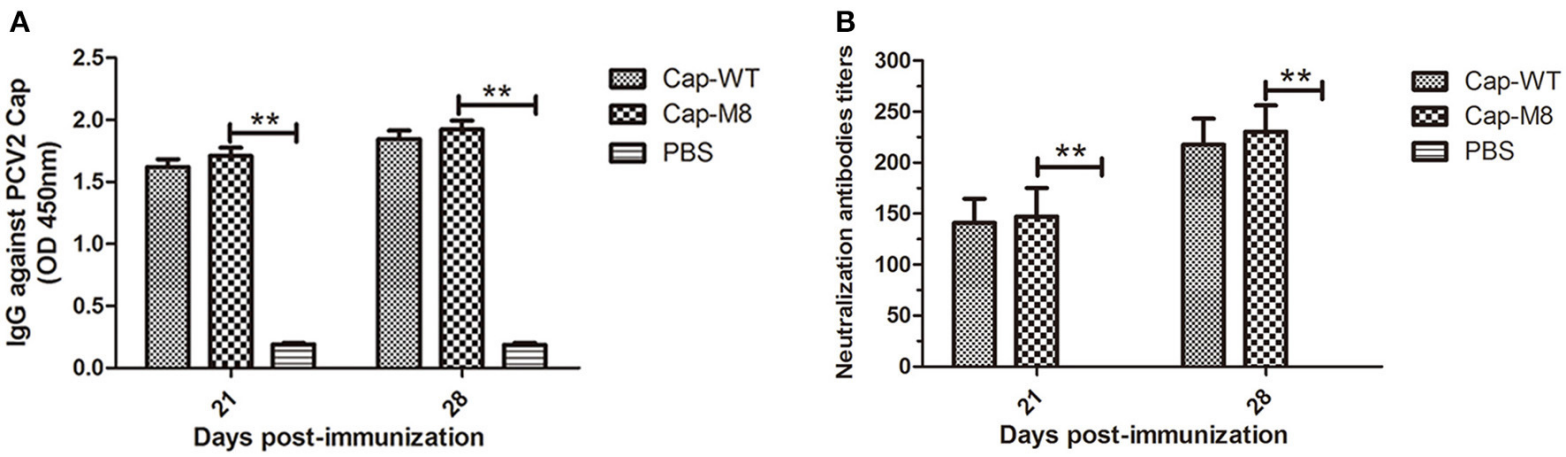
Humoral immune responses to VLPs assembled from PCV2 Cap and Cap-M8. **(A)** Cap-specific antibody in pigs detected by indirect ELISA. Sera were tested for presence of IgG antibodies by indirect ELISA at 21 and 28 d post immunization. PCV2 VLPs were used as an antigen for the ELISA. Optical densities were read at 450 nm. **(B)** Neutralization activity was evaluated by NAbs titers in pig sera. Bars represent arithmetic means ± *SD* of antibody titers. ^**^P < 0.01.

Although replacement of MP-Lcd with the foreign peptide was not detrimental to PCV2 VLPs assembly and cell entry *in vitro*, effects of foreign peptide on immunogenicity of PCV2 VLPs had not been determined. Therefore, pigs were immunized with VLPs assembled either from the Cap-WT or the Cap-M8. Anti-PCV2 antibody was determined by indirect ELISA at 21 and 28 d post immunization (dpi). Antibody titers increased rapidly (*P* < 0.01) from 21 to 28 dpi, compared to the control group (Figure 6). Based on ELISA, PCV2 Cap-specific antibodies were successfully induced by PCV2 VLPs assembled from Cap-WT or from the Cap-M8, with no statistical difference in titers (Figure 6A). Further, PCV2 NAbs were detected at 21 and 28 dpi, and both anti-PCV2 Cap sera had neutralizing activity against PCV2 infections *in vitro*, whereas no NAbs were detected in the PBS group (Figure 6B). Notably, both anti-PCV2 Cap sera had consistent virus-neutralizing titers, which strongly indicated replacing MP-Lcd with the foreign peptide had no adverse effect on eliciting PCV2 Cap-specific NAbs in pigs.

## Discussion

Inserting a foreign peptide into the middle of a protein often poses a huge challenge, as this may adversely affect protein folding, and thereafter affect protein functions. Although foreign peptides or tags are usually fused to the NT or CT of proteins to minimize effects on protein backbones, there is no assurance that inserted foreign peptides or tags will be displayed on the surface of proteins or protein complexes (i.e., VLPs). However, PCV2 VLPs have been developed to carry foreign peptides, with fusion usually done on either the NT or the CT of the Cap (Li et al., 2013; Zhang et al., 2014). Since the NLS at the NT of the PCV2 Cap is not required for VLPs assembly (Khayat et al., 2011), several PCV2 cVLPs were successfully prepared by replacing the NLS with a T-cell epitope, B-cell epitope, or a T-cell epitope conjugated with a B-cell epitope of classical swine fever virus (CSFV). However, no neutralizing antibodies against CSFV were detected (Zhang et al., 2014). Perhaps these foreign epitopes fused at the NT of the PCV2 Cap were buried inside the PCV2 VLPs after assembly. However, an epitope located on the exterior surface of VLPs may be presented to B lymphocytes by binding to B cells receptor (BCR) (Bachmann and Jennings, 2010). Conversely, the CT of the PCV2 Cap is exposed on the surface of PCV2 VLPs (Khayat et al., 2011; Liu et al., 2016; Wang et al., 2016), and was used to fuse with foreign peptides, although no typical VLPs were observed by TEM (Hu et al., 2016). We also failed several times with fusion of distinct tags to the CT (data not shown). In our previous study, Loop CD exploited a target site to insert a B-cell epitope derived from PRRSV GP5 protein, and the recombinant Cap (rCap) was capable of self-assembling into VLPs *in vitro* (Hu et al., 2016). Furthermore, cVLPs entered PK15 cells and induced NAbs against PCV2 and PRRSV infections. Loop CD of PCV2 Cap has several advantages over other loops of this protein for insertion of a foreign epitope (Hu et al., 2016). Recently, a novel genotype of PCV, named PCV3, was reported, although its pathogenesis and lesions remain to be determined (Phan et al., 2016; Palinski et al., 2017). Despite amino acid sequence identity between the Caps of PCV2 and PCV3 being only ~30% (Palinski et al., 2017), the PCV3 Cap had a similar jelly-roll as the PCV2 Cap, present in many icosahedral viruses (Khayat and Johnson, 2011; Zhan et al., 2017). Surprisingly, MP-Lcd was missing in the PCV3 Cap (Figure 1A), which indicated this portion may not be necessary for Cap folding into a jelly-roll structure and VLPs assembly during PCV evolution. Loop CD of the PCV2 Cap is the third largest loop of the Caps with 18 residues and forms the second highest elevation on the capsid surface (Khayat et al., 2011). Furthermore, MP-Lcd in Loop CD contributes the entire plateau portion to the two-fold axis surface of the PCV2 capsid (Figure 1D). Simulated 3D structures of the PCV2 cVLPs assembled from the Cap mutants (M1-6) clearly demonstrated that MP-Lcd functioned as a platform to display foreign peptides outward (Figures 3, 4, red). Of note, Loops CD of two neighboring Cap subunits were tightly aligned through their anti-parallel arrangement on the PCV2 capsid surface (Wang et al., 2016). Thus, two MP-Lcd were exactly side by side (Figure 1D, red), providing potential bivalent binding sites between foreign peptides (ligands) and their binding partners (receptors). Moreover, foreign peptides caused minimal alteration to the surface of PCV2 VLPs, since they projected from the plateau and were independently displayed. Thus, these foreign peptides might have no negative effect on assembly and antigenicity of PCV2 VLPs. Consequently, conformations of foreign peptides on the plateau also increased the likelihood of interactions with binding partners, e.g. BCR. By virtue of the MP-Lcd orientation within the PCV2 capsid, this is a promising target site for display of foreign peptides. In addition, retaining the plateau of MP-Lcd, instead of deleting it, projects foreign peptides further from the surface of the PCV2 cVLPs, based on the simulated 3D structures.

Further, we tested whether MP-Lcd was biased regarding amino acid composition of foreign peptides. Four PCV2 Cap mutants (Cap-M1 to M4) containing insertions with distinct foreign peptides were capable of self-assembly into VLPs *in vitro* (Figure 3B), and these VLPs retained their capacity to enter PK15 cells (Figure 3C). In particular, insertion of either poly-lysine or poly-glutamic acid residues into MP-Lcd (Cap-M3 and M4) had no effect on VLP assembly and cell entry (Figures 3B,C). Therefore, foreign peptides with highly charged residues may adopt very flexible conformations on MP-Lcd to minimize effects of charge repulsion on VLPs assembly. It also proves MP-Lcd exhibits high tolerance to insertions of various peptides. Indeed, 3D structure simulation also suggested both insertions independently protruded from the surface of MP-Lcd on PCV2 VLPs (Figure 3). Finally, maximal insertion size of MP-Lcd was tested. Two PCV2 rCap containing two distinct epitopes or three repeats of the same epitope in tandem, respectively, were successfully expressed and purified (Figures 2B,C). Morphologies of both assemblies had different sizes after rCap were dialyzed in assembly buffers (Figure 4B). Therefore, we concluded that morphology of PCV2 VLPs may be changed when the insertion size exceeded 18 residues in MP-Lcd. However, morphologies of PCV2 VLPs may also be changed *in vitro* through alterations of assembly conditions (e.g., temperatures and buffer), as reported for other VLPs (Tsukamoto et al., 2007).

Roles of MP-Lcd in PCV2 VLPs assembly, cell entry, and antigenicity were also investigated in the present study. Apparently, removal of MP-Lcd caused the plateau to disappear from the surface, but had no effects on VLPs assembly and cell entry. Therefore, we inferred that MP-Lcd may not be involved in recognition of virus-host cells for PCV2 internalization. Further, deleting MP-Lcd and replacing it with a foreign epitope (Cap-M8) introduced a new surface pattern around the two-fold axes of the PCV2 VLPs, since part of residues of the foreign epitope were present on the surface (orange in Figure 5A). Results from EM and IFA suggested that this Cap mutant was still capable of self-assembling into VLPs and entering PK15 cells. We also used this mutant to test effects of surface alteration around the two-fold axes on antigenicity of PCV2 VLPs in a swine model. Based on ELISA and neutralization experiments, we inferred that removal of MP-Lcd and alteration of the plateau around the two-fold axes of PCV2 VLPs had minimal effects on antigenicity and eliciting NAbs against PCV2 infection.

In conclusion, the MP-Lcd of Loop CD was identified as a very promising site within the PCV2 Cap to develop a functional carrier capable of displaying a variety of foreign peptides or epitopes on the surface of PCV2 VLPs. Of note, it was dispensable for assembly and cellular uptake of PCV2 VLPs. In addition, MP-Lcd was not required for eliciting NAbs against PCV2 infection. Therefore, development of PCV2 VLPs-based bivalent or multivalent vaccines against concurrent infections of PCV2 and other pathogens should be very feasible. Furthermore, based on structure simulation, MP-Lcd functioned as a plateau to project foreign peptides from the surface of PCV2 VLPs. Finally, although insertion size was tested, maximal size for MP-Lcd insertion still needs to be determined, as conditions may be further optimized for VLPs assembly *in vitro*.

## Author contributions

DW, NW, and YY are responsible for experimental design. DW, SZ, YZo, WY, YJ, YZh, and YD did experiments, collected and analyzed data. DW and YY wrote the manuscript. All authors read and approved the final manuscript.

### Conflict of interest statement

The authors declare that the research was conducted in the absence of any commercial or financial relationships that could be construed as a potential conflict of interest.
